# Therapeutic Value of Certain Remedies

**Published:** 1876-09

**Authors:** H. E. Woodbury

**Affiliations:** Washington, D. C.


### THERAPEUTIC VALUE OF CERTAIN REMEDIES.

BY H. E. WOODBURY, M.D., WASHINGTON, D. C.

*Helonias Dioica*, (*False Unicorn*).—This is a remedy that we believe has been too much neglected by the profession. Its use, so far as we can learn, has been confined almost exclusively to eclectics. It is without doubt the best of all the uterine tonics, proving most valuable in all of those diseases that have their origin in atony of the uterus. In all such cases we order it with great confidence, using it in all those cases of uterine ulceration where local applications are required. The best preparation, probably, is the fluid extract. Of this the dose varies, some of the manufacturers making it stronger than others. Of Tilden's, the dose is from dr. j to dr. ij. Of Sharp & Dohme's, from dr. ss to dr. j. It has served us well for many years in the treatment of amenorrhœa, dysmenorrhœa, leucorrhœa, etc. Its administration, in proper doses, is never attended with any unpleasant results.

We ordered this remedy some years since for a lady who was about to leave the city for the summer. She was suffering from endometritis, a case of long standing, complicated with enlargement and prolapsus. We had an opportunity to make only three or four local applications in this case, but enjoined upon the patient the necessity for taking the helonias as directed. Shortly after she left we received a letter from her, asking for the prescription, as she had used all the medicine, and "had never taken anything that had benefitted her so much," etc., etc. She informed us, on her return from New Jersey, that she had to send to Philadelphia (thirty miles) to get the recipe filled.

In the United States Dispensatory (edit. 1858, pg. iij), a brief reference (of seven lines) is made to this important medicine, taken from the Boston *Medical and Surgical Journal*. This, with a few sentences in Tilden's *Supplement to the Journal of Materia Medica*, is all the literature we have seen regarding it.

In our article that appeared in the *Medical Monthly* for July, 1876, a misprint occurs. We do not use the fluid extract of hedeoma, but the fluid extract helonias dioica, as a uterine tonic. The mistake undoubtedly resulted from the fact that the hedeoma, or pennyroyal, is much better known than the helonias dioica, the false unicorn.

*Sulpho-Carbolate of Sodium.*—We know of but two physicians who have used this remedy besides ourself—an article that we consider one of the most valuable in the entire materia medica. Our attention was first directed to it by an article in one of the journals in 1869. At that time we were treating a bad case of scarlet fever. We tried in vain to procure the sulpho-carbolate of sodium; it could not be found in our city at that time. Before it was received from New York our patient died. Since that time we have had many cases to treat, sometimes three or four in the same family in succession. In every case the sulpho-carbolate of sodium has been commenced early, and continued until convalescence was assured. Of course this was not entirely depended upon, but the local lesions and other indicatious were met, as seemed necessary in each individual case. A brief history of one of our cases may prove of interest.

W. C., aged four years, the child of parents who were of robust constitution, was seen by me, late at night, suffering from a burning fever, face flushed, skin dry and rough, pulse 130. Warm teas were freely administered during the night, with spts. ætheris nitrosi. The next morning the eruption was well developed, and a clear diagnosis (scarlet fever) was made. Sulpho-carbolate of sodium was at once ordered, and the bowels moved by a mild laxative. On the third day anginose symptoms of a severe type appeared. The throat was thoroughly mopped *ter in die*, with a solution of potas. permanganat. dr. j to O j of water, and the patient was ordered to take a little beef tea daily, if it did not disturb the stomach. In accordance with an old fancy, to please the parents, bacon rind was applied to the child's neck. The case proved to be a severe one, as the following sequelæ proved. About the twenty-fourth day the glands on both sides of the jaw began to swell. By the use of tinct. iodini, locally, the swelling on one side was arrested, but finding that the other side was not affected by its application, we poulticed it freely, and when soft opened the abscess that had formed. After the fever had run its course, there was such a debility that when the little patient attempted to drink from a cup his hand shook like that of a person affected with palsy. This recovery, though slow, was complete.

In order to form a correct idea of the gravity of a case of scarlet fever, Trosseau, in his *Clinical Medicine*, vol. II, p. 195, says, we must bear in mind the type of the prevailing epidemic. When we treated this case the epidemic was of a severe type, not less than half a dozen deaths having occurred in the neighborhood, while many of those who recovered suffered from incurable sequelæ. Our little patient is, to-day, entirely free from the lesions that so often follow this terrible disease. We say terrible, for before we used the sulpho-carbolate, we confess that we felt unable to cope with it successfully. Since we have been using it, we have not lost a single case.

In rubeola we have found it equally efficacious. In one family we had three cases of the disease—a child, its mother and uncle. In the child and uncle the disease was of a mild type. In the mother it was of a malignant character—the worst case we ever saw. Her friends and neighbors called it "a case of black small-pox." The eruption seemed of a livid color. So severe was the cough that we feared pneumonia would complicate the case. To our surprise this result was prevented. From the commencement the sulpho-carbolate of sodium was administered, and this, with expectorants, laxatives, etc., carried our patient safely through.

In several cases of variola and varioloid, we have had excellent results from the use of this remedy, not having lost a single patient from these diseases since we have been using it.

We would, by no means, give the impression that we depend upon this remedy entirely in these cases; far from it. Yet we use it persistently—believing, as we do, that it acts in such a manner as to materially modify the poison in these diseases of a zymotic type, and aids in the elimination of the poison from the system. Any peculiar symptoms or complications that may arise, we meet with such remedies as the nature of the case may seem to require.

As a rule, no unpleasant effects follow its administration, and large doses are well tolerated. An adult may take from grs. xx to dr. j *ter in die.* To children we give from grs. x to xv, dissolved in aqua cinnamon. They generally take it without difficulty, as the taste is not offensive. If we may base our opinion of the merits of the sulpho-carbolate of sodium upon an experience of over six years' employment of it in all of our cases of a zymotic type, we must candidly confess that we believe it to be a remedy of superior value in such cases, one well worthy of a more general trial by all who desire to "try all things and hold fast that which is good."—*Virginia Med. Monthly.*

